# Ultra-Wideband Radar-Based Indoor Activity Monitoring for Elderly Care

**DOI:** 10.3390/s21093158

**Published:** 2021-05-02

**Authors:** Matti Hämäläinen, Lorenzo Mucchi, Stefano Caputo, Lorenzo Biotti, Lorenzo Ciani, Dania Marabissi, Gabriele Patrizi

**Affiliations:** 1 Centre for Wireless Communications, University of Oulu, 90570 Oulu, Finland; 2 Department of Information Engineering, University of Florence, I-50139 Firenze, Italy; lorenzo.mucchi@unifi.it (L.M.); stefano.caputo@unifi.it (S.C.); lorenzo.biotti@unifi.it (L.B.); lorenzo.ciani@unifi.it (L.C.); dania.marabissi@unifi.it (D.M.); gabriele.patrizi@unifi.it (G.P.)

**Keywords:** home, living, movement identification, remote monitoring, signal classification, *k*-nearest neighbour

## Abstract

In this paper, we propose an unobtrusive method and architecture for monitoring a person’s presence and collecting his/her health-related parameters simultaneously in a home environment. The system is based on using a single ultra-wideband (UWB) impulse-radar as a sensing device. Using UWB radars, we aim to recognize a person and some preselected movements without camera-type monitoring. Via the experimental work, we have also demonstrated that, by using a UWB signal, it is possible to detect small chest movements remotely to recognize coughing, for example. In addition, based on statistical data analysis, a person’s posture in a room can be recognized in a steady situation. In addition, we implemented a machine learning technique (*k*-nearest neighbour) to automatically classify a static posture using UWB radar data. Skewness, kurtosis and received power are used in posture classification during the postprocessing. The classification accuracy achieved is more than 99%. In this paper, we also present reliability and fault tolerance analyses for three kinds of UWB radar network architectures to point out the weakest item in the installation. This information is highly important in the system’s implementation.

## 1. Introduction

According to demographic prognosis, it is evident that the ratio between the number of groups of people who need different kinds of care services, such as the elderly and minors, and those of working-age, is increasing globally [1]. That requires changes to existing care procedures but also requires utilization of new technology and data analytics in a different manner to make this fundamental change in care pathways possible. For example, using Statistics Finland’s information, it can be estimated that the ratio between ‘age group 0–14 & 65+’ and ‘age group 15–64′ will increase from ~61% in 2019 to ~66% in 2035, and to ~70% in 2050 [2]. This means that the same ratio, if compared only to the number of healthcare professionals, is radically much higher, which means increasing pressure loads on healthcare systems and professionals in the future.

Modern technology enables us to use new healthcare processes, and it also incorporates those from outside caring institutions, such as hospitals, elderly care homes, etc. At the same time, more responsibility will be given to people themselves. Society is moving towards individualized and personalized healthcare, where more emphasis is placed on prevention rather than acting after any symptoms are noticed. Currently, it is already possible to perform various reliable health-related measurements and diagnostics at home. Collected data can then be automatically transferred and stored electrically to patient and other records in the cloud, from where authorized medical staff can gain access to those data. Different kinds of wearables and carry-on medical devices will help in data collection and make it independent of location. In addition, modern smart phones have applications (apps) which monitor, for example, a person’s activity, their steps, etc. Active engagement with measurements could be easy for young and working-age people. However, in the case of elderly people, who might have dementia, or some other condition which decreases their physical mobility, wearables are not a good solution for self or remote monitoring. Carry-on devices, even though they would be easy and comfortable to use, at least in principle, require active commitment from the user. Gadgets can be left behind, and their batteries require constant changes or charging, which could make them inefficient in the long run. Being too complicated to use affects those devices which are not necessarily utilized, even though the benefits would be high. Especially in elderly monitoring, high usability is one of the major features for achieving user acceptance.

Instead of using wearable or other carry-on devices to detect and monitor a human’s vital signs, activity or presence, the use of devices embedded to the environment could be more favourable. Mains-connected devices do not require battery charging, which simplifies their usage. This is especially important in cases when remotely monitoring an elderly person’s activities or some of their vitals in their normal living environment is required. As such, reliable remote monitoring also enables elderly people to live longer in their own homes rather than being hospitalized or moved to another residence. Thus, the impact of bringing technological solutions to homes is two-fold: providing a better living quality for a person, and a reduction in institutional healthcare costs. In this paper, we discuss a technique which does not need any carry-on devices but is embedded to the environment and, thus, is seamless for the end-user while still providing desired information.

Enabling accurate movement or posture recognition requires good spatial resolution for the hardware used. As time and delay domain resolutions are inversely proportional to signal bandwidth, ultra-wideband (UWB) technology is favoured for this kind of solution. The other advantage of UWB in the healthcare domain is its very small transmission power, which decreases the power spectral density to the level of a noise floor. Thus, the UWB signal is safe for the user from the exposure point-of-view [3]. The other benefit of using UWB signal is its capability to be operated in an underlay fashion, thus coexisting with other radio systems sharing the same spectrum.

One field where UWB radar can be used in the healthcare domain is, for example, contactless respiratory and heartrate detection, which requires a good time domain resolution. This information can be squeezed out from the detected UWB radar signal, as the very wide bandwidth enables high resolution to distinguish tiny body movements. The other use-case is detecting whether somebody is in a certain space, such as a room. Using only one UWB device, it is possible to detect a person(s) presence, movement, and some of their vital signs, all at the same time. However, the latter option typically requires a static environment and a rather short distance between the UWB radar and its object, due to the small power levels used. Advanced data postprocessing techniques will also allow for the enabling of measurements in non-static cases. The use of UWB radar technology does not need a line-of-sight view either, which improves its usability for through-the-wall type measurements.

### 1.1. State of the Art

Exploiting UWB radars in movement detection is not a novel idea, and it has been previously widely studied. In this paper, we are not aiming to present an exclusive literature review, but to point out some of the corresponding work during recent years. This state-of-the-art (SotA) discussion directs our work towards our solution. For example, in [4], a UWB radar is used to distinguish whether a person in a room is moving, in a fixed position, or if the room is empty. The authors applied machine learning (ML) during their data analysis. Detection of multiple targets in static environment using UWB radar is also studied in [5], where detection is based on the calculation of correlation functions between time-domain responses measured from an empty room and from a room which has a person(s) inside. The authors showed that they are able to localize multiple persons in a space, but only in a static environment. Similar results are presented in [6]. Multiple persons’ tracking and human gait analysis using UWB radar is discussed in [7]. In that study, data analysis was based on the Doppler characteristics of the received signal. Using spectrograms, the authors were also able to calculate both respiration rates and walking patterns. Extracting respiration rate from the radar echo when person is moving is a harder task to perform due to the small signal levels received and relatively small chest movements. However, the authors concluded that it is attainable in the data postprocessing phase. Multi-object recognition in wider scope, associated with a data fusion and IoT, is discussed in [8].

In [9], UWB radar was used to detect and classify posture and motion changes using twelve predefined activities. It was also demonstrated that, by using UWB radar, it was possible to detect human walking by through-the-wall measurements, and also estimate range of movement [10,11]. The latter paper presents a method which is based on using one transmit–double receive technique. In [12], data processing is used to distinguish twelve individual gaits and five sequential gaits using frequency modulated continuous wave radar and three UWB radars simultaneously. In this case, UWB signal detection is based on analysing the identifiable Doppler shift patterns caused by the different gaits. In [13], deep learning is utilized in data postprocessing to help distinguish humans, three different animals, and empty room situations using through-the-wall UWB radar. The authors mentioned that the proposed technology can also be used in rescue applications, e.g., after earthquakes, to localize buried persons. One innovative application where UWB radar can also be used is in people counting. Due to the good resolution UWB radar has, individual persons can be separated from the received probing signal. This solution is presented in [14], where convolutional neural networks were used to improve counting accuracy.

UWB technology also enables contactless measurements of human vital signs. As mentioned above, extremely wide bandwidth makes it possible to detect small movements, e.g., to detect heartrate or breathing. Moreover, comparative analysis between UWB radar and three lead electrocardiograms (ECG) was performed in [15] to demonstrate that UWB radar can also be applied to ECG measurements. Authors of [16] used a living dog in their experiments to detect respiration via non-contact UWB radar. Contactless respiratory and heartrate measurements are also discussed in [17], where both line-of-sight and through-the-wall experiments are presented. In [18], authors proposed an algorithm to improve reliability to simultaneously detect respiratory and heartbeat rates using contactless UWB radar in the case of multiple targets and low signal-to-noise ratio conditions. Authors in [19] proposed a method to detect multiple persons through-the-wall by detecting their respiratory rates using fast Fourier transform and variance statistics. Permutation entropy and ensemble empirical mode decomposition-based algorithms for detecting respiration and heartbeat signals are developed and tested in [20]. The measurements were carried out in a static environment.

Similar kinds of measurement that our paper discusses are shown in [21], where UWB radar and data analysis using range scan are performed. Four different activities were identified in real-time. The authors envisioned in their paper that, in further studies, they will have a similar goal of merging vital sign and presence detections in a similar way to how we discuss in our paper.

Not only applicable in residency environments, UWB radar is a feasible solution in rescue and other safety related operations, as presented in [13,22]. The dual use of analysis of the received signal can be utilized to observe and locate living persons inside ruins, an avalanche, etc. That would be a lifesaving feature in emergency situations, as rescue efforts can be correctly directed. However, this use-case is out of the scope of this paper, but it expands the utilization of UWB radars in another safety critical domain. 

Automatic classification of movements has been studied in recent years, once the Internet-of-Things (IoT) and, in particular, wearable sensors became a reality. In [23], a survey of deep learning techniques for classifying movements is reported. In [24], several ML techniques to support human activity recognition are investigated. The previous papers are based on the use of wearable sensors. Many users, particular elderly users, do not find it easy to wear something all day long. In addition, ageing of individuals is often associated with cognitive impairments, which make it even more difficult to remind them to wear something, even though this is for their own health.

The use of a radar in the environment of human activity recognition has recently been proposed as a solution for the abovementioned problem. In [25], discrimination between movements and static positions is proposed by applying statistical moments (Skewness, Kurtosis, etc.) to the incoming frequency-modulated continuous wave (FWCW) radar signal. In [26], only Kurtosis is used for the detection of human movements with a UWB impulsive radar. In [27], a bi-static UWB radar, together with two video cameras, is used to provide movement classifications. However, the use of video cameras might cause an ethical issue and, thus, it is not the best option if a person’s privacy needs to be protected. In [28], a set of UWB radars in a 40 square meters apartment were used to provide movement recognition, supported by an ML technique. Although accuracy reached 80%, multiple radars were required to achieve this performance. In our study, we propose a solution which is very simple, friendly for the user, and easy to implement in the home, while still providing good accuracy.

### 1.2. Our Contribution

In this paper, we present a method which uses only one UWB radar device for multiple detection purposes, and which simplifies detecting a person’s presence and detecting some pre-selected movement patterns. Moreover, remote detection of breathing and coughing can also be performed using the same installation. Later on, we show that, by utilizing connected UWB radars, it is possible to cover larger areas, such as a residence with several rooms. UWB radar-based monitoring is ethically viable, as it does not use or transfer any information identifying a person, such as video streams, and it does not require any user registration. Thus, there is no need to collect any personal information. Moreover, in this paper, we represent a fault tolerance and reliability analysis for the studied system, which, according to our knowledge, has not been performed before for this kind of approach. The main contributions of this paper can be summarized as follows:

- Single UWB radar used to identify dynamic and static postures of the user in a room;
- Investigation of the possibility to extract breathing and coughing rates when the user is in a static posture;
- Selection of three simple statistical parameters to discriminate between static postures;
- ML technique, called as *k*-nearest neighbour, to automatically classify static postures;
- Reliability and fault tolerance analysis for the radar device and a home network;
- Network architecture solutions proposed to support such home networks in a large-scale deployment.

The rest of the paper is organized as follows. Section 2 describes the devices, experimental set-up and data analysis used in our experiments. Section 3 is dedicated to reliability and fault tolerance analyses related to the networked UWB radar concept. Section 4 introduces the network architecture options to implement a flexible UWB radar monitoring network in a home environment. The results achieved and discussion are provided in Section 5. Finally, Section 6 concludes the paper.

## 2. Experimental Set-Up

### 2.1. UWB Radar System

**Figure 1:**
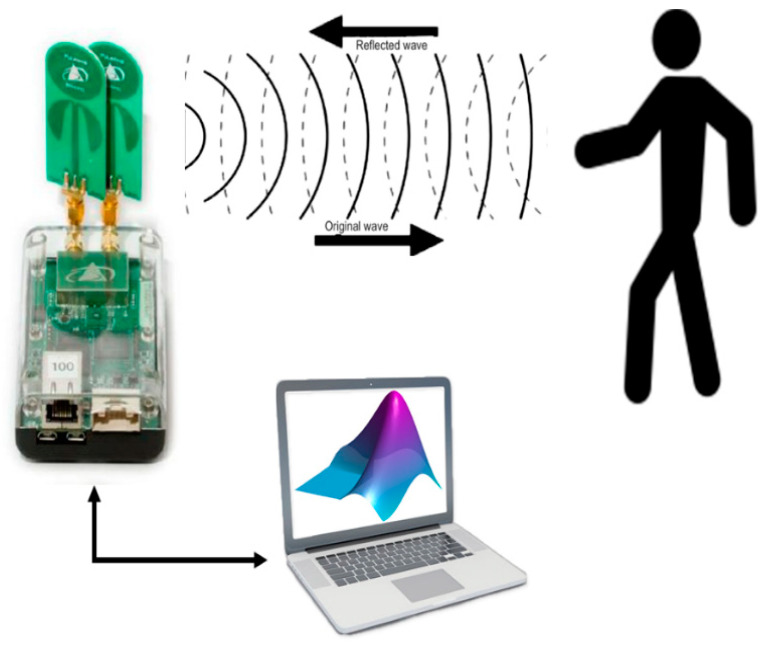
UWB radar module and experiment setup.

In this section, we describe the UWB radar system used in our study. All experiments were carried out using the Time Domain Corporation’s PulseOn P440 UWB Radio Module [29] in a monostatic radar mode. The UWB radar used covers a frequency range from 3.1 GHz to 4.8 GHz. The radio frequency (RF) emission, having maximum power spectral density of −41 dBm/MHz, is compliant with both the United States Federal Communications Commission (FCC) Part 15 and UWB regulations [30] and the European Union ETSI EN 302 065 [31] standard’s radiation mask. Both antennas (transmitting and receiving) are Time Domain Broadspec Toroidal Dipole Antennas and have a gain of −3 dBi [29]. In a horizontal plane, the antenna provides omni-directional transmit and receive patterns, supporting a frequency range of 3.1–5.3 GHz, thus covering the frequency band of the transmitted signal. The transmit power is 50 µW. The pulse integration index was set to 15, thus providing a 45 dB processing gain at the receiver (10*log_10_2^15^ ≈ 45). During the experiments, the UWB radar module was connected to a MATLAB customized application running on a PC for data visualization and analysis purposes, as illustrated in Figure 1. The line-of-sight coverage of the UWB radar module is ~80 m for a walking person, which is more than needed to detect and track a person in a typical residential room.

### 2.2. Experimental Studies

**Figure 2:**
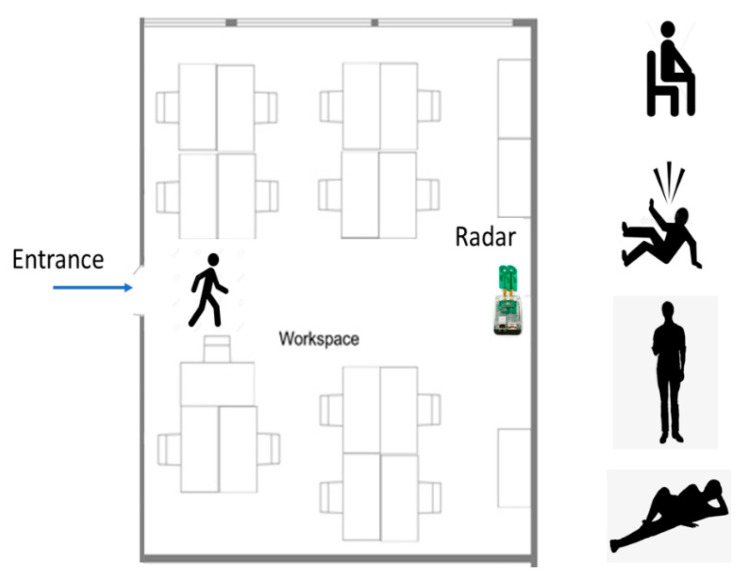
Experimental setup at the university office room.

Experiments were carried out in an office room at the University of Florence. The room dimensions were 4 m × 5 m × 3 m. The UWB radar was put on a wall at a height of 1 m, on the opposite side of the entrance (see Figure 2). The room had typical office furniture (desks, chairs, scaffolds, etc.), which correlate with the furnishing of a sanitary environment. One chair was put in the middle of the line between the entrance and the radar. Experiments had the objective of providing enough data to develop an algorithm for the classification of selected movement actions: walking, standing up, sitting down, lying down, and falling. In addition, the entrance and exit of a person to and from a room was also provided by the same data, thus indicating when a person was present in a room. 

A volunteer made different movements from the entrance of the room towards the radar and back, 10 times, to accumulate data. Only voluntary researchers were involved in the experiments. Informed consent was obtained in advance from these people. The movements made by the volunteer were the following:

- Walking from the entrance towards the radar and back.
- Walking from the entrance to the chair and sitting down for a while, then standing up and walking back to the entrance.
- Walking from the entrance to the middle of the room and lying down (slowly) for a while, then standing up and walking back.
- Walking from the entrance to the middle of the room and falling.

Additionally, the possibility of remotely detecting coughing and breathing was investigated while keeping the volunteer in a selected posture; they were sitting on a chair, standing up or lying down on the floor.

### 2.3. Data Analysis

After the experiments, the recorded data was postprocessed to achieve the desired information. In particular, raw data coming from the UWB radar module was stored onto the PC and then postprocessed in MATLAB. The raw data was first bandpass filtered to fit the UWB regulation mask and mitigate noise. Then, data was analysed to identify and classify activity/movement patterns. To decide which type of activity could be associated to the person in the room, different techniques can be used, from maximum likelihood (MLi) to machine learning (ML) [23]. Usually, ML is preferred since it does not require a mathematical reference model (e.g., backscattering propagation from a human body) to be used. On the other hand, ML techniques require a learning and configuration period whose duration and repetition frequency affect to the classification performance. 

The approach presented in this paper was aimed at keeping the monitoring system as simple as possible, not only from the cost to the elderly point-of-view (by using only one radar sensor per room), but also in the view of data analysis. An empty room radar snapshot was recorded and used as a reference template. In addition to the hardware installation itself, this was the only action required to perform before the system was ready for use. In data postprocessing, the recorded template was subtracted from the radar swaps of the room, which were measured during the presence of the individual. This allowed us to distinguish the responses between the empty room and the movements/positions of the individual inside that room.

The UWB radar scanned the room from the distance range of 3.1 m to 4.3 m over time. This distance interval was selected for the analysis of the echo. Let *r*_n,k_ be the *k*-th received sample of the *n*-th scan as

(1) $$r_{n,k}=s_{n,k}+w_{n,k}\;,$$

where *s_n,k_* is the desired reflected sample and *w_n,k_* is the noise sample. The *N* × *K* matrix ***R*** is acquired and processed using MATLAB. In our case, number of sweeps *N* = 500 and number of sweeps *K* = 96. The sample bin length is 42 ps, which equals to 1.25 cm in distance. The swap time, thus the time between a scan and the next one, is 30 ms.

A previously recorded matrix of the empty room ***R***^(empty)^, a reference template, is subtracted from the result matrix ***R*** in order to isolate the samples with the presence of the body in the room as

(2) $$X\;=\;R\;-\;R^{\left(\mathrm{empty}\right)}$$

Statistical parameters applied to the steady positions will help in identifying body position. In particular, we have seen that statistical parameters of skewness and kurtosis, together with power, can be used to discriminate between standing up, sitting, and lying postures. Skewness, kurtosis, and power are calculated as [32]

(3) $$Skewness=\frac{\frac{1}{K}\sum_{k=1}^{K}{\left({\bar{x}}_{k}-\mu \right)}^{2}}{\sigma^{2}}$$

(4) $$Kurtosis=\frac{\frac{1}{K}\sum_{k=1}^{K}{\left({\bar{x}}_{k}-\mu \right)}^{4}}{\sigma^{4}}$$

(5) $$Power=\frac{1}{K}\overset{K}{\underset{k=1}{\sum }}{\bar{x}}_{k}^{2}$$

where

(6) $${\bar{x}}_{k}=\frac{1}{N}\overset{N}{\underset{n=1}{\sum }}x_{n,k}$$

is the average over time of the elements *x_n,k_* of the matrix **X** defined in Equation (2), μ and δ are the mean and the standard deviation of ${\left\{x_{k}\right\}}_{1}^{K}$, respectively.

As shown during the postprocessing phase, respiratory activity and coughing can also be extracted by analysing the data during a steady position (standing, sitting, lying). In particular, the sitting position seems to be the best position for the extraction of breathing and coughing, as the human body is in a perpendicular position to the coming UWB signal and, thus, maximizes the radar cross-section of the body.

**Figure 3:**
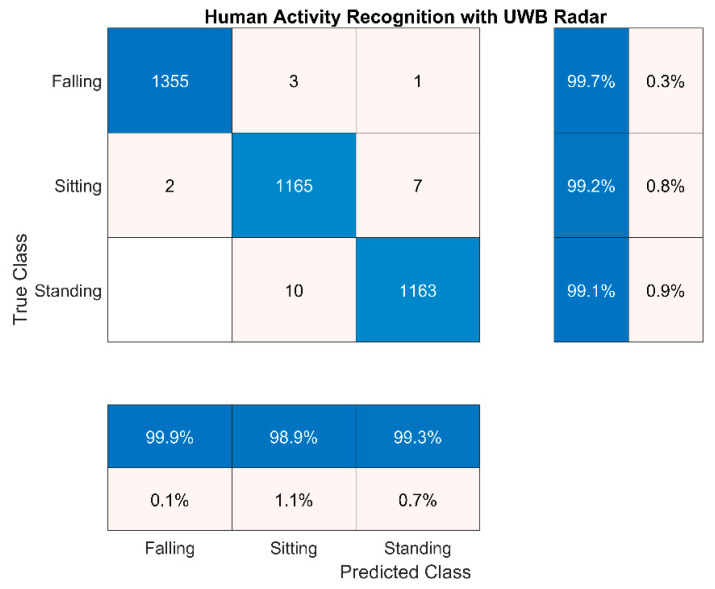
Confusion matrix for the ML KNN algorithm to classify the static postures.

In addition, we implemented an ML technique to classify the static posture automatically. In particular, the *k*-nearest neighbours (KNN) algorithm [33] was used to identify/categorize the static postures by analysing the radar signal. The incoming radar signal was firstly de-noised by subtracting the empty room snapshot. Then, the KNN ML algorithm was run by considering the three statistical parameters (3), (4) and (5) applied to the de-noised signal envelope. The results are shown by using the confusion matrix in Figure 3.

ML algorithm used for classification typically “learns” a pattern into part of the data set. Then, the rest of the data set is used to test the accuracy of the classification. In our case, we used 50% of the data set to train the ML algorithm, and the remaining part to test the accuracy of the classification. As it can be seen from Figure 3, the KNN ML algorithm was able to correctly identify the posture with an accuracy greater than 99%. This accuracy value is similar (sometimes even better) to those mentioned in the literature, e.g., [34,35]. In [34], a Doppler UWB radar and an ML technique were used to discriminate movements from fixed position, and accuracy spanned from 91.7% to 100%. In [35], the UWB radar, together with a convolutional neural network (CNN), was used to discriminate walking and standing, with an accuracy of 99.7%. It is important to point out that here we not only discriminated movements from static positions but we also identified the types of posture: standing, sitting, and falling/laying down.

## 3. Reliability Analysis and Fault Tolerance

This section provides a reliability evaluation of a network composed of UWB radars located in several rooms of a house. The Reliability Block Diagram (RBD) method was implemented to analyse the impact of different redundancy configurations in terms of reliability performance. The analysis was conducted in compliance with the international standard IEC 61078 (2016) [36] and the American standard MIL-HDB 338B [37].

The use-case and aim of the network is to monitor the activities of an elderly person living alone on 24/7 basis. As described in Chapter 1, an increasingly ageing population will lead to more efficient utilization of tele-monitoring to support and assist elderly people in their homes, as well as to try to decrease accesses to hospitals or other sanitary places. The sustainability of the public healthcare system is bonded to the performance and efficiency of modern and reliable e-health tele-monitoring services. More and more often, elderly people live alone in the contemporary era, particularly in western countries. The possibility of fully trusting a tele-monitoring system is the basis for keeping an eye on the elderly and to reassure their families. The need for quick reactions in the case of a health emergency is another key element to reduce the risk of serious or permanent health problems.

Reliability is a key feature in this kind of remote monitoring system. Thus, we are raising some key research questions to be solved. Supposing that the house of an elderly individual is provided with a UWB radar network consisting of one radar per room, and a gateway to accumulate and send data towards the cloud server. The question is, how secure and trustworthy is this kind of system? How can we guarantee that the fault of one device does not imply a failure of the entire service? The success of such a service is linked to the capacity of the network to provide fault tolerance, but how can we obtain it? 

Safety-critical applications, such as the one studied in this paper, require a deep study of system reliability and fault diagnosis. To ensure proper monitoring of the elderly person living alone, reliability analysis and redundancy optimization play a fundamental role. They allow us to identify design weaknesses and implement design strategies and countermeasures to maximize system availability and minimize the risk associated with the failures.

One of the most important design solutions for safety-critical systems is the implementation of fault tolerant architectures to ensure that the system continues its regular operations after an item fails. Fault tolerance is based on the use of one or multiple redundant items that cover the functionalities of the main element and guarantee continuity of service in the case of failure. In IoT applications, recent developments in semiconductor technologies have led to a fast increase in the use of low-cost, low-power multifunctional solutions. As a matter of fact, low-cost and low-power sensors allow an easy implementation of redundancy and, thus, fault tolerance in the network architecture can be improved.

A preliminary risk analysis according to the international standard ISO/IEC 31010 (2019) [38] is mandatory to draw attention to the main criticality of the developed system. Then, multiple Monte Carlo simulations have been run to obtain the RBD of the optimal architecture for the network under test. 

**Figure 4:**
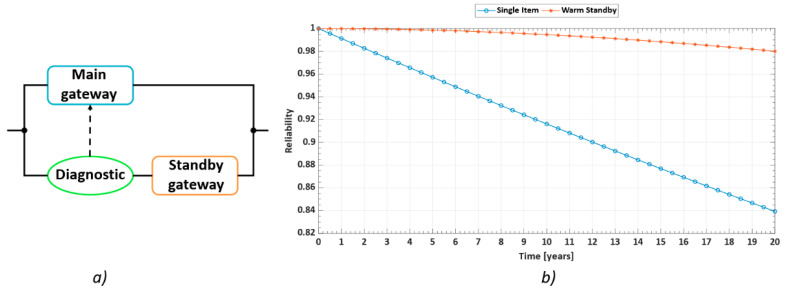
Proposed solution to achieve gateway fault tolerance: (**a**) illustrates the Reliability Block Diagram of the warm standby configuration.; (**b**) comparison between the reliability trends of a single gateway (blue curve) and of a fault tolerant warm standby comprising two gateways and a diagnostic system (red curve).

A risk analysis pointed out that the gateway is the most critical component within the network for two reasons. First, it is the most complex item and, thus, it is the most likely to fail, and second, it has to manage entire network functionalities and upload all measured data to the cloud. Therefore, in case of a failure of the gateway, data storage will be impossible, and the entire system will stop working. A “static” redundancy (i.e., the classical parallel architecture) cannot be implemented because of many problems: it is a fault masking technique without a proper fault detection; network status is not monitored and, thus, users are not aware of failures; a solution with two gateways working at the same time is unreasonable, as it consumes resources. Therefore, the solution to ensure continuity of the service and guarantee an extended useful life is the implementation of a warm standby redundancy. In this configuration, in case the active gateway fails, a diagnostic system called as a switch unit detects the failure and reconfigures the architecture, with the activation of a standby module maintaining the operation of the entire network [39]. This solution allows us to significantly improve network reliability, since the standby unit has a substantially reduced failure probability during the quiescent time. In fact, if the item is not working, the probability that it will fail is negligible. Figure 4a shows the warm standby architecture proposed for the gateway of the developed network. Monte Carlo simulations were run to estimate system reliability (as shown in Figure 4b), considering the time interval from 0 to 20 years. The results explicitly show the superiority of the warm standby architecture compared to the use of only one gateway in the system. Simulation parameters were estimated by means of the reliability prediction handbook Telcordia SR 332—Issue 4 [40], which is specifically developed to estimate the failure rate of telecommunication equipment.

The predicted failure rate of the active gateway is 1 failure per million hours (FPMH), while the standby gateway is characterized by a lower failure rate of 0.1 FPMH due to its inactivity in normal conditions.

**Figure 5:**
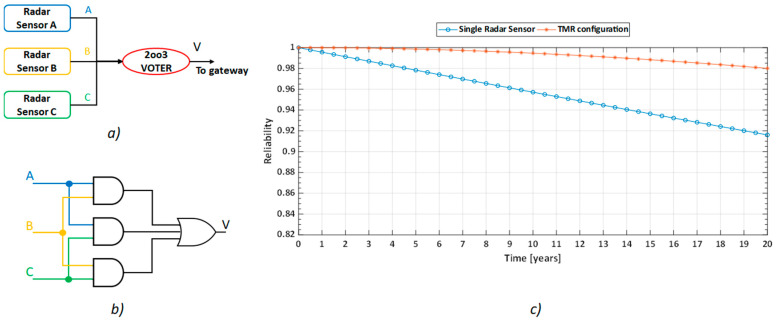
Proposed solution to achieve hardware fault tolerance for the radar sensor in each room: (**a**) RBD of the proposed TMR architecture; (**b**) logical scheme implementation of the majority-voting system in case of digital output of the sensors; (**c**) comparison between the reliability trends of a single radar sensor (blue curve) and of a fault tolerant architecture comprising 3 radar sensors (red curve).

Regardless of the use of a fault tolerant gateway system, it is extremely important to ensure that redundant data comes from the sensors in each room. For this reason, a fault tolerant configuration has been developed also for the radar sensors installed in the various rooms of the house. Supposing that three sensors were installed in each room, a possible solution would be to use a Triple Modular Redundancy (TMR), in which all three sensors acquired data at the same time. Then, the sensors’ outputs are processed by a majority-voting system called Voter, which is able to produce a single output according to the three inputs. TMR is an advanced version of 2-out-of-3 (2oo3) architecture [37], in case the Voter is not failure-free. If one out of three sensors in each room fails, then the Voter is able to generate a single output according to the response of the other two sensors. Therefore, when one of the three sensors fails, the outcomes of the other two are used to correct and mask the fault. Obviously, the most critical element of a TMR architecture is the majority-voting system because, in case of failure of this component, the benefits of redundancy will be nullified. For this reason, a reliability assessment of the Voter should be carried out carefully in order to ensure high reliability values of this component and to guarantee that the network will work as required. However, increasing the number of sensor nodes in a room, or in the entire residence, increases installation costs and reduces system simplicity. Figure 5 shows the proposed solution to achieve hardware fault tolerance for the three sensors installed in each room. Figure 5a shows the RBD of the TMR architecture. A possible solution to implement the 2oo3 majority-voting system (red circle in Figure 5a) is illustrated in Figure 5b, while, in Figure 5c, the reliability of the TMR fault tolerant architecture is compared with the reliability of a single sensor. In compliance with reliability handbook Telcordia SR 332–Issue 4 [40], the failure rate of a single radar sensor has been set to 0.5 FPMH. Again, it is evident that redundancy helps to significantly improve system reliability.

## 4. Network Architecture

This section presents the overall network architecture needed to develop a flexible and scalable remote health monitoring system based on UWB radars or corresponding sensing devices. A pervasive healthcare system aims at providing care services without any limitations caused by distance or time. Therefore, to have a large-scale deployment of indoor UWB radars’ networks, these must be integrated with terminals and wireless network technologies to support early diagnosis, real-time monitoring, and medical emergency management and alerting.

UWB radars used are able to identify the posture and motion changes of a person, and also to measure human vital signs. However, they require an elaboration of the collected data to extract useful information, storage of the data in a remote-control center, and an eventual alarm generation when needed. Thus, radars must be connected to the internet to fully benefit from the data they provide. Toward this goal, the IoT paradigm represents an efficient solution. Moreover, thanks to new wireless communications standards it is possible to provide high reliability and security with a reduced latency to carry patients’ health data efficiently using commercial systems.

The communications/computing system has to satisfy several functional and service requirements.

Functional requirements:

- Creating a resilient communications network with redundant coverage connected to the internet.
- Assuring security of access and data.
- Collecting data from radars and real-time analysis.

Service requirements:

- Supporting sufficient data rate: each radar generates about 250 kbps.
- Supporting reliable and low-latency data transfer.
- Providing sufficient computational capabilities: the proposed ML algorithm requires a computational complexity that is O(l*m*d), where *m* is the number of training elements, *l* is the number of voting neighbors and *d* is the dimension of the observation to be classified.

The aim of this section is to analyse possible enabling technologies/architectures that allow us to satisfy such requirements.

Two network architectures can be envisaged for both communications and computing: the remote-control center can be reached by means of a hierarchical infrastructure or directly. In any case, radars must have two communications interfaces: the UWB-interface for monitoring purposes and a wireless communications interface for transferring data.

In the hierarchical architecture, radars are connected wirelessly to a gateway that collects and locally processes acquired data. The gateway is capable of producing instantaneous monitoring results that can generate real-time alarms and provide data, which can be stored in the central cloud. The gateway can be a dedicated device connected to the remote-control center wirelessly or via wired connection. Alternatively, the monitoring application can be deployed on the patients’ personal device (e.g., tablet or smartphone). Communications then occur by means of personal/local area networks, such as Bluetooth (IEEE 802.15.1), ZigBee (IEEE 802.15.4) or Wi-Fi (IEEE 802.11).

In the second architecture, radars are directly connected to the cellular network and send their raw data to the control center using, for example, 4G/5G technologies. In this case, data are centrally elaborated in the cloud, where they can also be stored and used by the authorized persons.

The first solution is the most straightforward. It allows the UWB radars to have limited communications capabilities (i.e., short range), and the gateway enables an instantaneous data processing to provide real-time information. However, as evidenced in Section 3, the gateway can represent the point of failure of the entire monitoring system; thus, a redundant architecture should be considered to assure service continuity with a very high probability. Moreover, the computational capabilities of the gateway can be limited, which reduces the amount of processing that can be locally performed for the collected data.

The direct connections of the radars require that all devices are connected to the cellular network, and data are centrally executed. In the cloud, there are more computational capabilities available, but higher latency is introduced in the response time. It is important to stress that, with the new cellular communications systems, strict reliability and latency performances should be guaranteed, thus also making it possible to provide critical services, such as the ones related to health monitoring and prompt intervention. Current 5G and future 6G systems can provide inherent security mechanisms to better protect data, and they also provide high-priority traffic and network slicing opportunities for better dedicated services [41].

**Figure 6:**
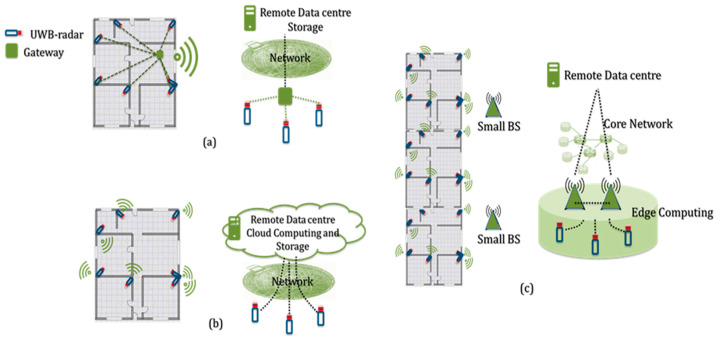
Overall network architecture: (**a**) hierarchical architecture; (**b**) UWB-radars directly connected; (**c**) architecture based on cellular features, such as 5G/6G.

The two hierarchical architectures are represented in Figure 6a,b. These architectures can converge, exploiting the new network paradigms that are arising with the 5G/6G communications systems, such as dense cell, or even ultra-dense, deployment and an edge computing [42]. The idea of network densification is to increase the number of access points reducing the cell size and drawing the transmitter and receiver closer to each other. In this way, dedicated capacity can be provided where needed. In the proposed system, a local pico or femto cell (i.e., a small Base Station—BS) can be used to provide UWB radars and access to the cellular network. This is beneficial not only in terms of dedicated capacity but also for security issues. Indeed, suitable strategies can be adopted for enabling access only to a limited group of devices, for instance. This is not possible using the traditional network architecture, where large macro cells provide connectivity to a multitude of heterogeneous users within a large coverage area.

Moreover, in the 5G/6G network, the concept of edge computing has been widely investigated. Computing can be moved to the edge of the network, thus providing computational capabilities near to the points where data are produced and can be locally utilized. Following this concept, small cells and UWB radars’ computational capabilities are pooled so that there is a sufficient processing capacity close to where it is needed. This architecture is presented in Figure 6c. This is particularly useful when UWB radars are used to monitor larger buildings, such as nursery houses, hospitals, or rehabilitation centers. One or more small BSs can provide connectivity and computation capabilities.

Finally, as stated before, security is a fundamental issue when personal or critical data are exchanged. For this reason, suitable data protection mechanisms should be adopted. The 5G/6G networks provide inherent security by design. However, security can be increased by adopting limited access strategies, and also integrating additional security approaches, i.e., cryptography, with low cost and low complexity physical layer security strategies.

## 5. Results and Discussion

This section introduces the results from an experiment mimicking a use-case where a single UWB radar sensor is used to provide information about an elderly individual who is living alone in their home. The information inferred, in our case, is mainly related to movements (walking in or out of the room, sitting down, standing, falling, etc.) Additionally, we wanted to check whether information about respiration and coughing during “steady” positions, i.e., standing up, sitting, lying down, could be extracted from the measured data.

**Figure 7:**
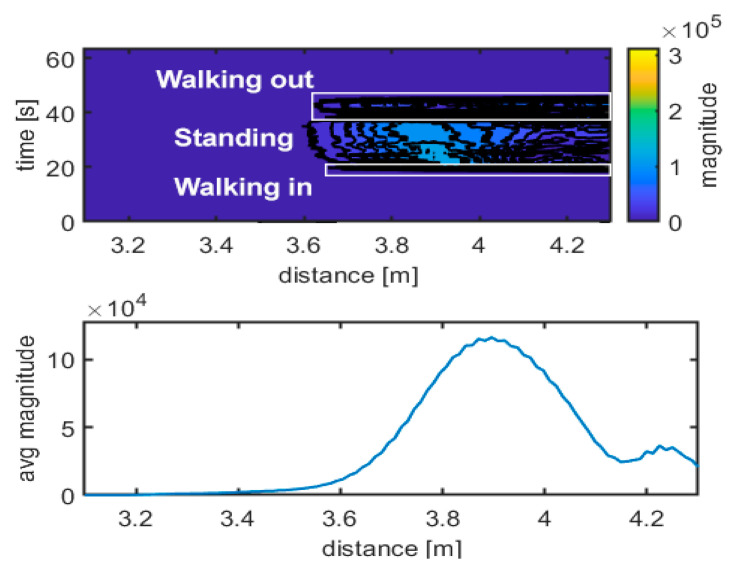
Movement identification. Experimental results: walking in–standing frontside–walking out. Magnitude unit is µV.

**Figure 8:**
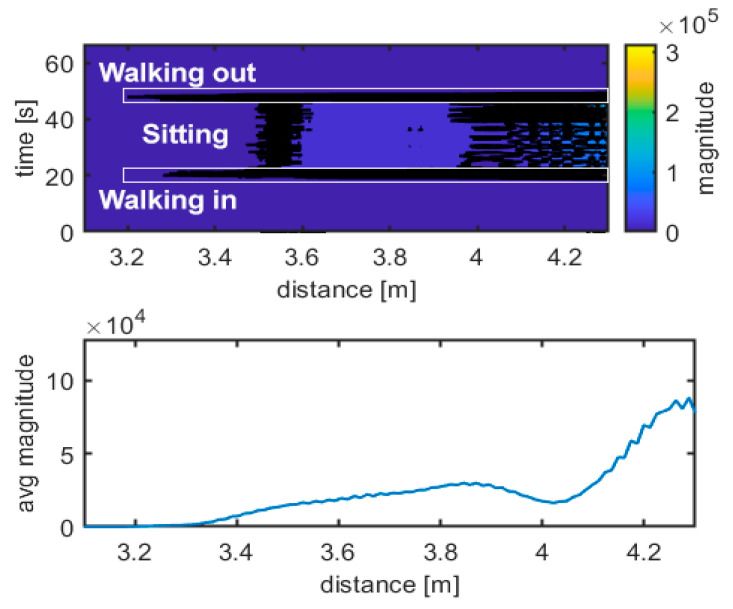
Movement identification. Experimental results: walking in–sitting frontside–walking out. Magnitude unit is µV.

**Figure 9:**
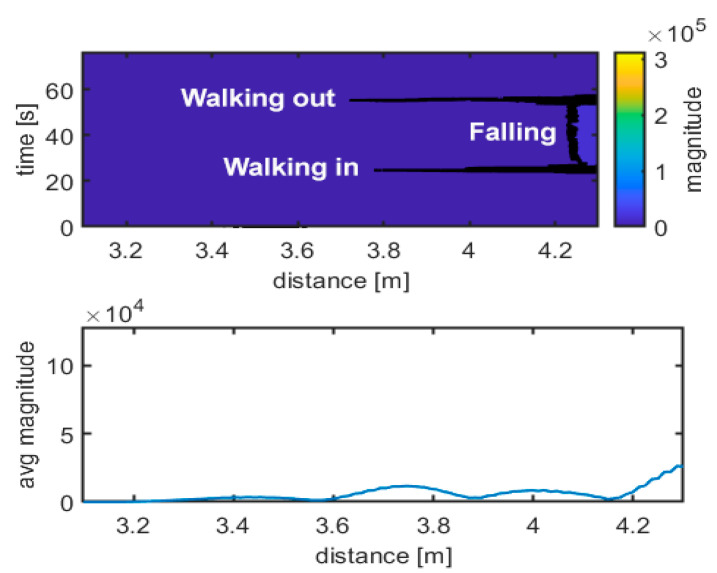
Movement identification. Experimental results: walking in–falling–walking out. Magnitude unit is µV.

**Figure 10:**
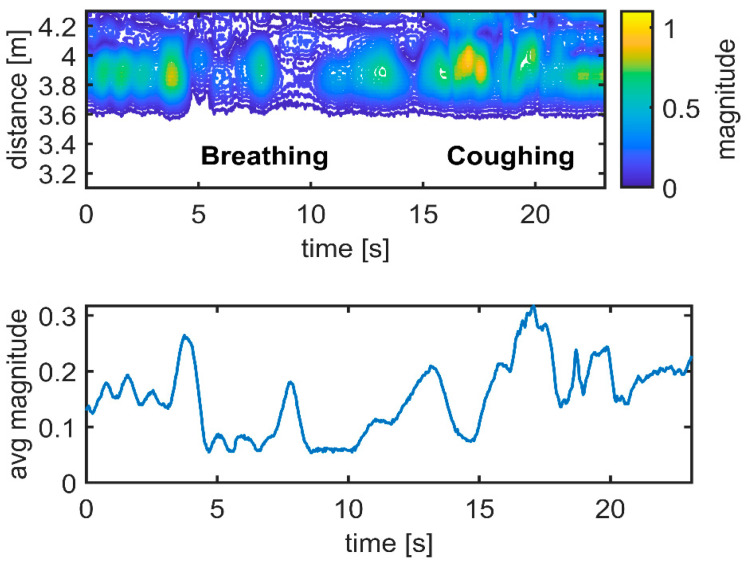
Respiration and coughing. Experimental results: standing frontside. Magnitude unit [µV] is normalized.

**Figure 11:**
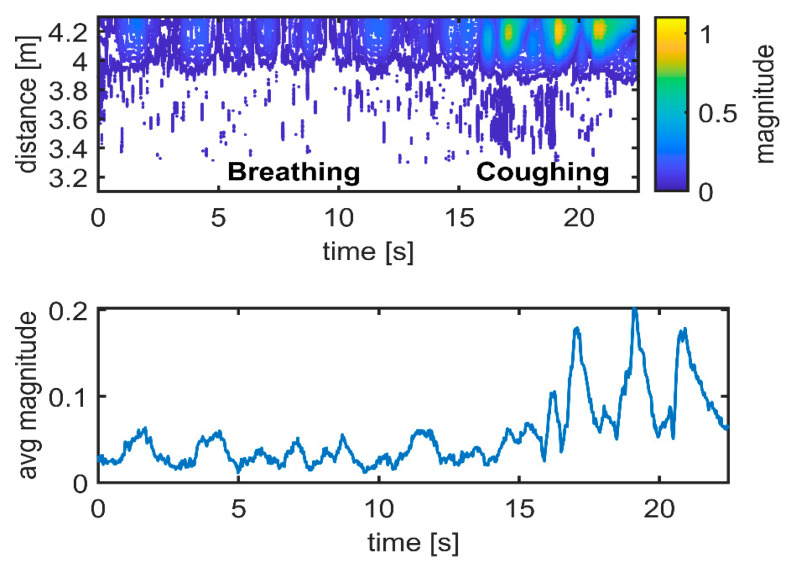
Respiration and coughing. Experimental results: sitting frontside. Magnitude unit [µV] is normalized.

The upper graph in Figure 7 shows the difference between the data acquired from the specific movement and the empty room snapshot (template), calculated as described in Equation (2). In this case, walking in, standing frontside and walking out maneuvers can be easily distinguished, and they are shown. The different scheme of the radar traces is evident. The lower graph in Figure 7 shows the received radar echo, extracted from data and averaged over time during a steady interval (no movement). The shape of the signal is characteristic of the standing up position. Figure 8, Figure 9, Figure 10 and Figure 11 follow the corresponding presentation format for different movements and postures.

Figure 8 and Figure 9 show the corresponding results for sitting and falling/lying postures, including walking in/out a room phases, respectively. The traces of walking can easily be identified compared to the steady positions (no movement). Doppler frequency can also be evaluated to identify movement intervals, direction, and steady intervals. It is more challenging to discriminate what the position of the body is during steady intervals. The radar echo, once subtracted from the empty room echo, can be used to discriminate between body positions during steady intervals, as the upper and the lower graphs in Figure 7, Figure 8 and Figure 9 show. Additional support to decide can come from a simple statistical analysis on the radar magnitude, averaged over time during the steady interval. Kurtosis is quite different in the cases of standing, sitting or lying, as depicted in Table 1. The power of the echo can also be used as a discrimination parameter; in particular, for power in the case of falling and for skewness in case of standing (Table 1). For example, the standing position seems to have the lowest skewness and kurtosis, together with the highest received power, while falling seems to have the highest skewness and kurtosis, together with the lowest received power.

**Figure 12:**
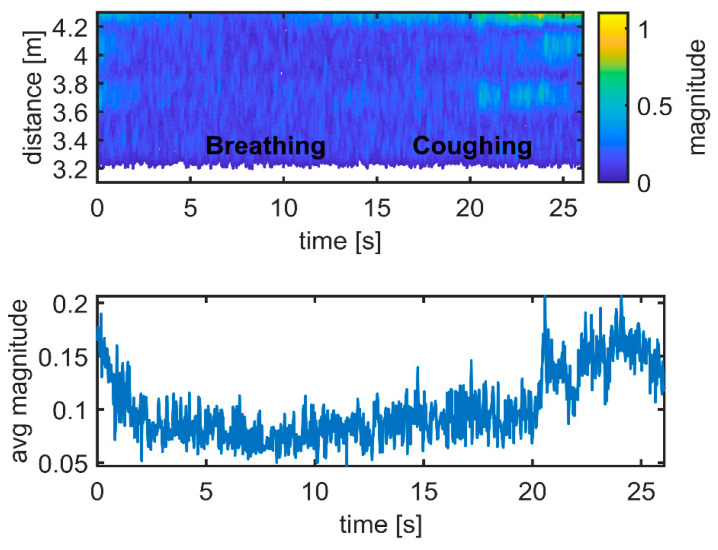
Respiration and coughing. Experimental results: lying down. Magnitude unit [µV] is normalized.

The upper graphs in Figure 10, Figure 11 and Figure 12 show the differences between the data and the empty room snapshot (Equation (2)) in a steady position (standing frontside, sitting, falling and lying down). Breathing and coughing patterns are extracted from the data. The magnitude of the processed data is here normalized to better highlight the peaks of breathing and coughing. In particular, the individual was breathing normally for 15 s (about 6–7 breaths) and then coughing for 10 s (about 5 coughs). The lower graphs in Figure 10, Figure 11 and Figure 12 show the received radar echoes averaged over time during the steady intervals (no movement) to measure breathing and coughing rates. The different scheme of the radar traces is evident, in particular when the individual is sitting. Breathing and coughing rates are not easily visible during standing (Figure 10), while they are evident during sitting (Figure 11). If the individual is lying down, it is not possible to extract breathing or coughing rates, as depicted in Figure 12, when exploiting the measurement setup used in this study (Figure 2).

## 6. Conclusions

In this paper, we have presented a remote monitoring architecture to seamlessly monitor elderly citizens in their homes utilizing ultra-wideband (UWB) radar as a sensing device. UWB provides good spatial resolution to contactlessly distinguish different body postures, presence, coughing, breathing, and other activities based on the trajectories of a moving person, or small movements of, for instance, a person’s chest. UWB is also a safe technology due to the very small power spectral density of the transmission; thus, it does not cause any harm to people. The major idea behind our experiment was to keep installation and data analysis as simple as possible. Compared to wearable sensors, the use of a single radar sensor embedded in the environment allows for simpler monitoring for the user, particularly for elderly users or for individuals with cognitive impairments.

In addition to discussing only posture monitoring, we present a reliability evaluation of the network composed of UWB radars disposed in several rooms of the house. Both hierarchical and direct cellular based concepts are covered.

This paper provides a comparative reliability analysis between three different UWB radar installations to connect to the internet: using one gateway only, one using redundancy provided by a warm standby-gateway, and one using a two-out-of-three voting-based setup. It is evident that any redundancy improves the network’s fault tolerance. As all network traffic passes the gateway, it is the most critical component in the home network, and it should be carefully selected to maximize the entire network’s reliability.

Using the experimental approach, we represented how to extract different movements (walking in and out, falling), steady positions (standing, sitting and lying), as well as breathing and coughing from the collected UWB radar data. Using very simple statistical analysis, such as skewness and kurtosis parameters, different movements/postures can be reliably discriminated. Breathing and coughing can be extracted with high quality during steady positions, particularly during sitting.

A *k*-nearest neighbour machine learning algorithm is used to automatically discriminate between and classify static postures. The ML algorithm is applied by using the three statistical parameters of the incoming radar signal’s envelope, once the empty room snapshot is subtracted from raw data. Classification accuracy is found always to be greater than 99%.

To increase the detection reliability for breathing and coughing, the use of more than one UWB radar in a room might be needed. Accuracy depends heavily on the position of a person in relation to the UWB radar. However, one device per room, as used in our study, is a reasonable number for an easy and cost-efficient implementation to detect larger movements, such as walking, falling or breathing/coughing during sitting periods, which are sufficient when monitoring an elderly person’s daily activities. As usual, there is a trade-off between implementation cost and achieved detection accuracy.

In future studies, we will expand UWB radar installation to residence level and analyse the performance of a larger monitoring network. To maintain the system’s simplicity, we are still sticking to one device per room setting.

## Figures and Tables

**Table 1:** Statistical parameters for movement identification.

Position	Skewness	Kurtosis	Power [µW]
Standing	0.83	2.26	2820
Sitting	1.44	4.64	1010
Falling	1.78	6.74	61
